# Molecular Characterization of *Paralichthys olivaceus* MAF1 and Its Potential Role as an Anti-Viral Hemorrhagic Septicaemia Virus Factor in Hirame Natural Embryo Cells

**DOI:** 10.3390/ijms22031353

**Published:** 2021-01-29

**Authors:** Julan Kim, Ja Young Cho, Ju-Won Kim, Dong-Gyun Kim, Bo-Hye Nam, Bong-Seok Kim, Woo-Jin Kim, Young-Ok Kim, JaeHun Cheong, Hee Jeong Kong

**Affiliations:** 1 Biotechnology Research Division, National Institute of Fisheries Science, Busan 46083, Korea; kimjulan@korea.kr (J.K.); jjy6556@naver.com (J.Y.C.); ogamzar@korea.kr (J.-W.K.); combikola@korea.kr (D.-G.K.); nambohye@korea.kr (B.-H.N.); bskim2002@korea.kr (B.-S.K.); yobest12@korea.kr (Y.-O.K.); 2 Fish Genetics and Breeding Research Center, National Institute of Fisheries Science, Geoje 53334, Korea; wj2464@korea.kr; 3 Department of Molecular Biology, Pusan National University, Busan 46241, Korea

**Keywords:** MAF1, *Paralichthys olivaceus*, fish, CRISPR/Cas9 system, VHSV

## Abstract

MAF1 is a global suppressor of RNA polymerase III-dependent transcription, and is conserved from yeast to human. Growing evidence supports the involvement of MAF1 in the immune response of mammals, but its biological functions in fish are unknown. We isolated and characterized *Maf1* from the olive flounder *Paralichthys olivaceus* (*PoMaf1*). The coding region of *PoMaf1* comprised 738 bp encoding a 245-amino-acid protein. The deduced PoMAF1 amino acid sequence shared features with those of MAF1 orthologues from vertebrates. *PoMaf1* mRNA was detected in all tissues examined, and the levels were highest in eye and muscle tissue. The *PoMaf1* mRNA level increased during early development. In addition, the *PoMaf1* transcript level decreased during viral hemorrhagic septicemia virus (VHSV) infection of flounder hirame natural embryo (HINAE) cells. To investigate the role of *PoMaf1* in VHSV infection, single-cell-derived *PoMaf1* knockout HINAE cells were generated using the clustered regularly interspaced short palindromic repeats/CRISPR-associated-9 (CRISPR/Cas9) system, and cell clones with complete disruption of *PoMaf1* were selected. *PoMaf1* disruption increased the VHSV glycoprotein (G) mRNA levels during VHSV infection of HINAE cells, implicating PoMAF1 in the immune response to VSHV infection. To our knowledge, this is the first study to characterize fish *Maf1*, which may play a role in the response to viral infection.

## 1. Introduction

MAF1, a central RNA polymerase (pol) III-associated transcription repressor, is highly conserved from yeast to human [1]. MAF1 was discovered in the yeast, *Saccharomyces cerevisiae* [2]. MAF1 represses RNA pol III in response to unfavorable growth conditions, such as oxidative stress, DNA damage, rapamycin or chlorpromazine treatment, and secretory pathway inhibition [3]. In mammals, MAF1 represses not only RNA pol III-dependent transcription, but also RNA pol II-dependent transcription of the transcription initiation factor, TATA-binding protein (TBP) [4]. Because TBP is required for the function of RNA pol I, MAF1 can impact transcriptional activity directly and indirectly [5]. Moreover, phosphorylated MAF1 is localized at the promoters of tRNA and 5S rRNA genes, and represses their expression [6].

Recent studies have focused on the physiological role of MAF1, mainly in lipid metabolism and tumorigenesis. MAF1 controls intracellular lipid accumulation and de novo lipogenesis by repressing transcription of the lipogenesis-related genes *FASN* and *ACC1* [7]. Whole-body knockout (KO) of MAF1 in mice confers resistance to diet-induced obesity and nonalcoholic fatty liver disease [8]. MAF1 also inhibits cancer proliferation by suppressing AKT-mTOR signaling through activation of PTEN transcription [9]. MAF1 inhibits ERK1/2 signaling, a key component of the MAPK signaling pathway [10]. mTOR, which interacts with MAF1, is targeted by pathogens to promote their replication and plays important roles in the innate and adaptive immune response [11]. However, whether MAF1 is involved in the immune response is unclear. In addition, despite the diverse functions of MAF1 in a range of species, the roles of fish MAF1 are unknown.

The olive flounder *Paralichthys olivaceus* is an economically important marine teleost in Korea, China, and Japan. Viral hemorrhagic septicemia (VHS) is an OIE-listed disease that causes significant mortality in farmed fish, including *P. olivaceus*, rainbow trout (*Oncorhynchus mykiss*), and a broad range of wild freshwater and marine species worldwide [12,13]. The causative agent of this contagious disease is viral hemorrhagic septicemia virus (VHSV), a negative-sense single-stranded RNA virus of the genus *Novirhabdovirus*, family *Rhabdoviridae* [14]. Despite much research, a VHS vaccine is not yet commercially available [15]. VHSV is sensitive to common disinfectants, but these do not inactivate the virus [16]. Studies aiming to improve the management of VHSV infection are underway.

The RNA-guided clustered regularly interspaced short palindromic repeats/CRISPR-associated 9 (CRISPR/Cas9) system enables genome editing in diverse species, including human, monkey, mouse, rat, fly, and zebrafish [17,18]. CRISPR/Cas9 enables genome editing to generate KO or knock-in models with higher precision and efficiency than other genome-engineering tools, such as zinc-finger nucleases and transcription activator-like effector nuclease [19]. The CRISPR/Cas9 system uses a single-guide RNA (sgRNA) complementary to the target DNA sequence, and the Cas9 nuclease-sgRNA complex is programmed to cleave the target genomic locus bearing a protospacer adjacent motif (PAM) [20]. Genome editing using the CRISPR/Cas9 system has been performed in a variety of fish species, including Atlantic salmon (*Salmo salar*), channel catfish (*Ictalurus punctatus*), common carp (*Cyprinus carpio*), red sea bream (*Pagrus major*), and *P. olivaceus*, as well as in fish cells in vitro [21,22,23]. CRISPR/Cas9-based genome editing enables functional discovery of genes and genetic improvement of aquatic animals [18].

In this study, we isolated *P. olivaceus Maf1* (*PoMaf1*) cDNA to assess the role of MAF1 in the teleost fish *P. olivaceus*. The ORF sequence of *PoMaf1* was characterized, and multiple alignments of the deduced amino acid sequences of PoMAF1 and MAF1 orthologues from various species were conducted. The tissue- and developmental stage-specific *PoMaf1* mRNA profiles were determined in *P. olivaceus*. The *PoMaf1* mRNA level was investigated in flounder Hirame Natural Embryo (HINAE) cells during VHSV infection. To explore the role of *PoMaf1* in VHSV infection in *P. olivaceus*, we generated single-cell-derived *PoMaf1* KO HINAE cells using the CRISPR/Cas9 system and evaluated the effect of *PoMaf1* disruption on VHSV replication and the expression levels of immune response genes.

## 2. Results and Discussion

### 2.1. Isolation and Characterization of Maf1 Gene from P. olivaceus

**Figure 1:**
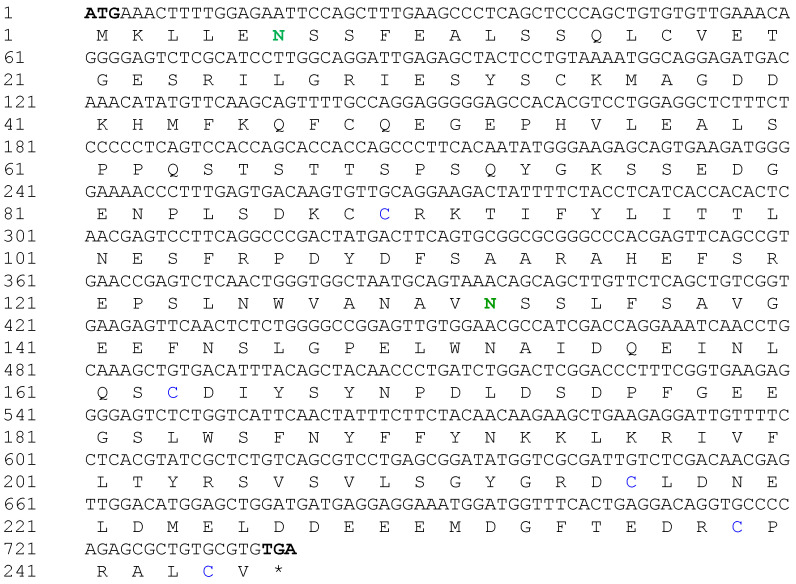
Coding sequence and deduced amino acid sequence of the *P. olivaceus Maf1* (*PoMaf1*). Start and stop codons in the coding region are in bold. Nucleotide and deduced amino acid residues are numbered to the left. Potential N-glycosylation sites are denoted in bold green. The predicted disulfide bond is indicated in blue.

The *PoMaf1* full-length coding region (738 bp) encoding 245 amino acids was obtained from *PoMaf1* cDNA (GenBank accession No. MW417124) (Figure 1). The putative molecular weight (MW) and isoelectric point (pI) of PoMAF1 were 27,805.74 Da and 4.39, respectively. Potential N-glycosylation sites and a disulfide bond were predicted in *PoMaf1*. The deduced amino acid sequence of PoMAF1 lacked a signal peptide sequence, such as a nuclear localization sequence (NLS). Yeast MAF1 has two conserved NLSs at the N- and C-termini (residues 205–208 and 328–332) [24]. In contrast, there is no canonical NLS in MAF1 of higher eukaryotes, including humans, similar to PoMAF1 [25]. MAF1 of all species localizes to both the nucleus and cytoplasm, and is expected to translocate into the nucleus as a negative regulator of transcription [26].

### 2.2. Amino Acids Sequence and Phylogenetic Analysis of PoMAF1

**Figure 2:**
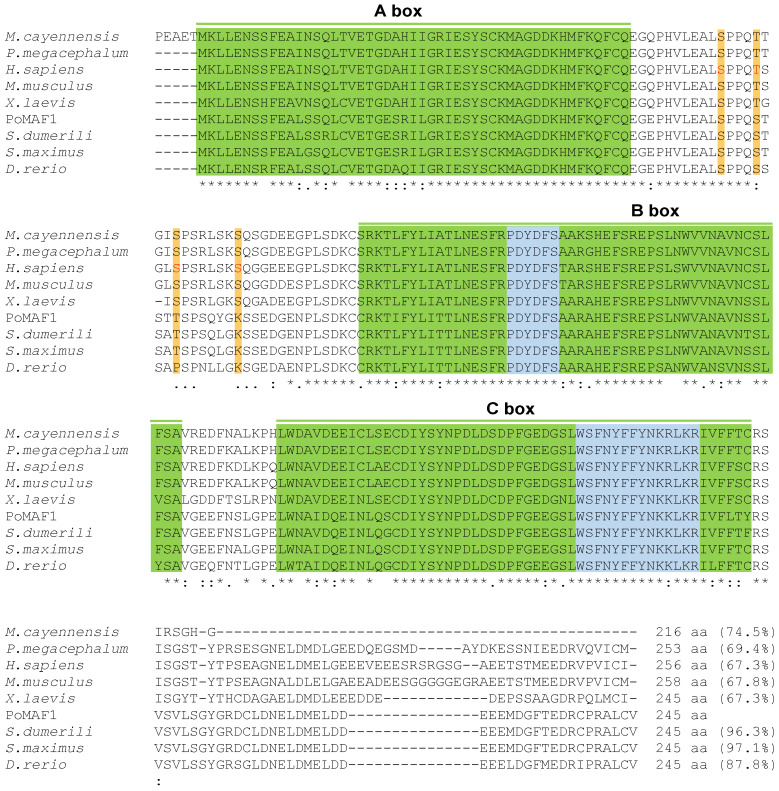
Alignments of the deduced amino acid sequence of *PoMaf1* with its orthologues in other vertebrates: *Mesembrinibis cayennensis* (NXL10327.1), *Platysternon megacephalum* (TFJ99060.1), *Homo sapiens* (AAH14082.1), *Mus musculus* (AAH16260.1), *Xenopus laevis* (NP_001080708.1), *Seriola dumerili* (XP_022622762.1), *Scophthalmus maximus* (XP_035474856.1), and *Danio rerio* (NP_001025410.1). Identical and similar residues are marked by asterisks (*) and colons (:), respectively. Residues that are somewhat similar are denoted by dots (.). A, B, and C boxes with high similarities in the MAF1 orthologues are boxed in green. The two MAF1 signature sequences, which are conserved in PoMAF1, are shown in blue. Thr and Ser residues that are phosphorylated in human MAF1 are indicated in red, and putative phosphorylated sites are boxed in orange. Similarity scores compared to PoMAF1 are shown at the end of the deduced amino acid sequences.

**Figure 3:**
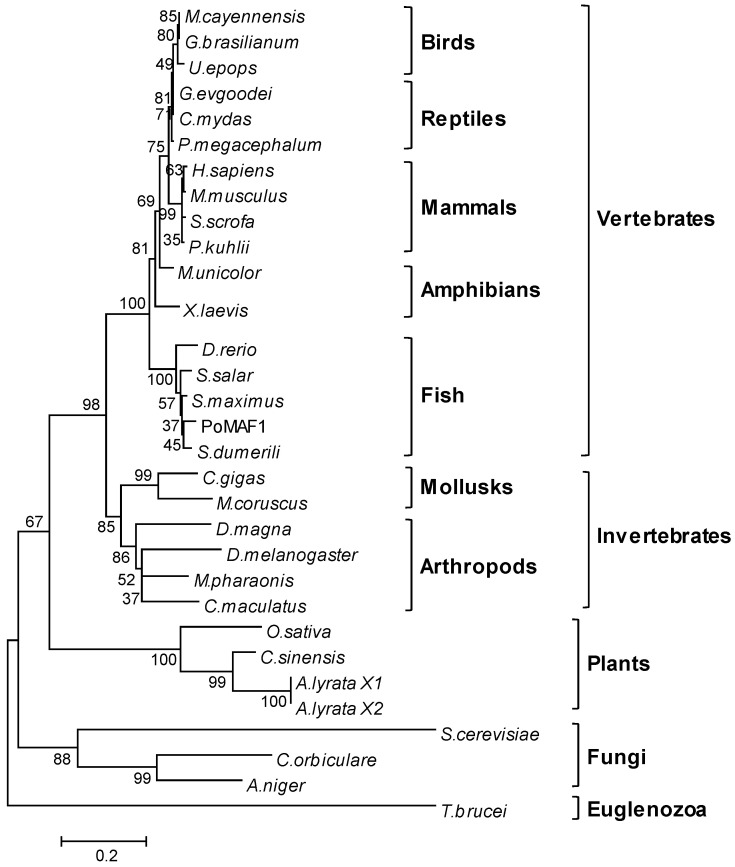
Phylogenetic relationship of PoMAF1 with its orthologues. The tree is based on alignment of the amino acid sequences from vertebrates—birds, reptiles, mammals, amphibians and fish—and invertebrates, and shows that PoMAF1 clusters with MAF1s from fish species. Bootstrap values are shown as percentages at the nodes. GenBank accession numbers of PoMAF1 orthologues are provided in Table 1.

The deduced amino acid sequence of *PoMaf1* was aligned with those of orthologues in mammals, reptiles, amphibians, birds, and fish (Figure 2). PoMAF1 exhibited the highest similarity (97.1%) with Black-Sea turbot *S. maximus*, a flatfish. The multiple sequence alignment revealed that PoMAF1 has three regions of high similarity to MAF1 orthologues: the A-, B- and C-boxes. The two MAF1 signature sequences (PDXDFS/T and WSXXYFFYNKKXKR) were present within domains B and C of PoMAF1, respectively. Phosphorylation sites (human residues Ser-60, Thr-64, Ser-68, and Ser-75) between the A- and B-boxes, which are directly phosphorylated by mTORC1 and required for mTORC1-mediated control of RNA pol III transcription, were found in PoMAF1 [27]. The A- and B-boxes are associated with RNA pol III subunits and Brf1, respectively [28]. The YSY motif (human residues 166–168) in the C-box of human MAF1 is critical for its stability and regulation [29]. Conserved elements in PoMAF1 suggest a role for PoMAF1 as a repressor of RNA pol III in *P. olivaceus*. Using multiple alignments, a neighbor-joining phylogenic tree was constructed based on the deduced PoMAF1 amino acid sequence and MAF1 sequences of 29 species (Figure 3). PoMAF1 was divisible into 10 main clusters: fish, amphibians, mammals, reptiles, birds, mollusks, arthropods, plants, fungi, and euglenozoa. PoMAF1 was grouped with orthologues from other teleost species and was most closely related to *S. dumerili* MAF1.

### 2.3. Tissue Distribution and Developmental Stage-Specific Expression of PoMaf1

**Figure 4:**
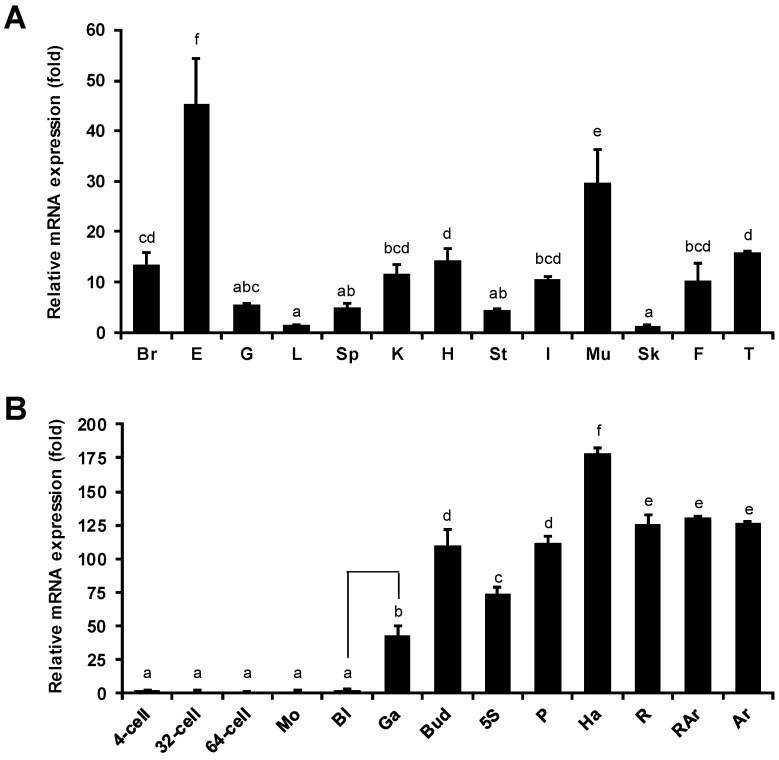
Tissue distribution and developmental expression of *PoMaf1* in *P. olivaceus*. (**A**) Tissue distribution of *PoMaf1* mRNA in *P. olivaceus*. The *PoMaf1* mRNA level was measured by quantitative real-time PCR. Values were normalized to that of 18S rRNA and are shown as fold relative values compared to skin. Br, brain; E, eye; G, gill; L, liver; Sp, spleen; K, kidney; H, heart; St, stomach; I, intestine; Mu, muscle; Sk, skin; F, fin; T, testis. (**B**) *PoMaf1* mRNA level during early development of *P. olivaceus*. Embryo and larvae samples were harvested from the four-cell stage to 27 days post-hatching (dph). mRNA levels were normalized to that of 18S rRNA and 64-cell-stage values were compared to those at other stages. 4-cell, 4-cell stage; 32-cell, 32-cell stage; 64-cell, 64-cell stage; Mo, morula stage; Bl, blastula stage; Ga, gastrula stage; Bud, bud stage; 5S, 5-somite stage; P, pharyngula stage; Ha, hatched larva (0 dph); R, rotifer feeding stage (12 dph); RAr, rotifer and Artemia feeding stage (20 dph); Ar, Artemia feeding stage (27 dph). Means and standard deviations of three independent replicates are shown. Means with the same superscript letter are not significantly different (*p* ≤ 0.05).

The mRNA levels of *PoMaf1* in 12 *P. olivaceus* tissues were determined by quantitative real-time PCR (Figure 4A). *PoMaf1* mRNA was ubiquitously detected in the tissues, and the highest levels were in eye and muscle tissue. In humans, the *Maf1* mRNA level is high in muscle [30]. In pigs, the mRNA expression profile of skeletal muscle changed in response to nutrient intake. MAF1 is a nutrient- and stress-sensitive global repressor of RNA pol III [31,32]. Therefore, MAF1 might be involved in the nutrient and stress responses of *P. olivaceus*. The high *PoMaf1* mRNA level in the eye suggests that PoMAF1 has diverse biological functions in various tissues.

The mRNA level of *PoMaf1* in *P. olivaceus*, from the four-cell stage to the feeding stage to 27 days post-hatching (dph), was evaluated by quantitative real-time PCR (Figure 4B). The mRNA level of *PoMaf1* remained low until the blastula stage, and increased 18-fold at the gastrula stage compared to the blastula stage. The *PoMaf1* mRNA level increased gradually and was highest in hatched larva. In the feeding stage, the *PoMaf1* mRNA level decreased by 70% and then remained constant. The *PoMaf1* mRNA level fluctuated markedly during early development, indicating a role for PoMAF1. MAF1 is a regulator of Akt/mTOR signaling in dendritic morphogenesis during neuron development [33]. This is in accordance with the marked increase in *PoMaf1* mRNA level during gastrulation, which is immediately followed by neurulation [34]. The expression profile of *PoMaf1* suggests that further investigation of the role of PoMAF1 is warranted.

### 2.4. VHSV Infection Decreases PoMaf1 mRNA Level

**Figure 5:**
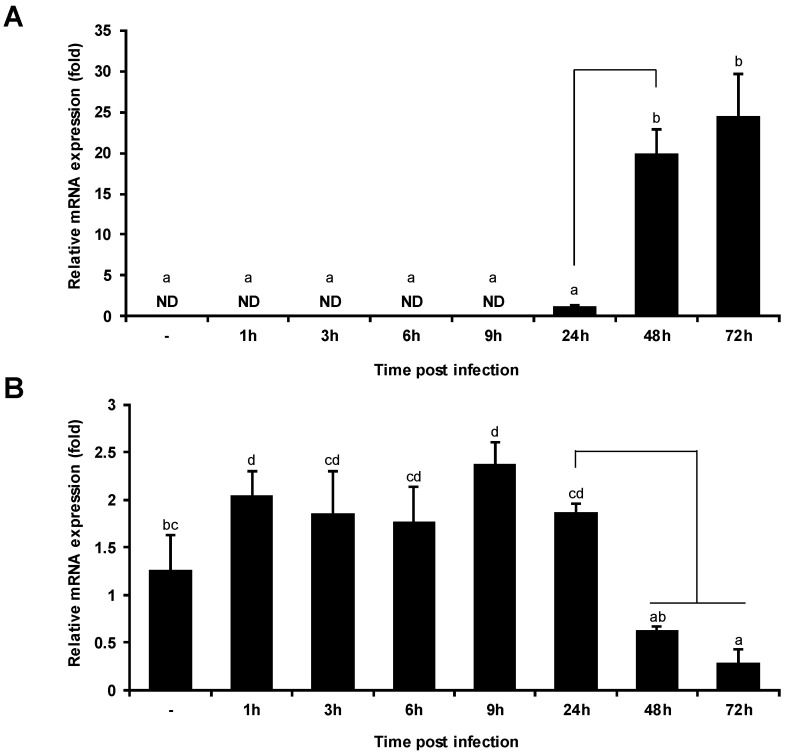
*PoMaf1* expression was reduced by VHSV infection in flounder HINAE cells in a time-dependent manner. (**A**) Transcript level of VHSV glycoprotein (G) in HINAE cells infected with VHSV. ND, not detected. (**B**) Transcript level of *PoMaf1* in HINAE cells infected with VHSV. HINAE cells were infected with VHSV at a MOI of 0.5 and samples were collected at the indicated time points. Transcript levels were analyzed by quantitative real-time PCR and are expressed as fold relative values over the control (0 h) normalized to 18S rRNA. Means and standard deviations of three independent replicates are shown. Means with the same superscript letter are not significantly different (*p* ≤ 0.05).

To determine the physiological relevance of VHSV and PoMAF1, quantitative real-time PCR of VHSV-infected HINAE cells was performed (Figure 5). During VHSV infection, the *PoMaf1* mRNA level increased moderately from 1 to 24 h, but was markedly decreased at 48 and 72 h. In addition, the VHSV glycoprotein (G) mRNA level significantly increased at 48 and 72 h post-infection. The decrease in *PoMaf1* mRNA level with increasing VHSV expression is in agreement with the postulated relationship between viral replication and MAF1 expression. MAF1 is a new target of PTEN that negatively regulates lipid metabolism, and Dengue virus (DENV) infection inhibits MAF1 via Akt/FoxO1/Maf1 signaling, which is mediated by PTEN, to regulate lipid metabolism and thus promote DENV replication [7,35]. Akt in the PI3K/Akt intracellular signaling pathway is activated in acute and persistent viral infection to suppress apoptosis and prolong viral replication [36]. The fish virus, *Siniperca chuatsi* rhabdovirus (SCRV), activates the PI3K/Akt pathway, thus inducing autophagy [37]. PI3K/Akt/FoxO1 signaling modulates MAF1 abundance, and PI3K/Akt/mTORC1 signaling regulates MAF1 in a posttranslational manner, such as by phosphorylation [38]. Therefore, *PoMaf1* expression might be related to the immune response to VHSV.

### 2.5. Disruption of PoMaf1 in HINAE Cells Using CRISPR/Cas9 System

**Figure 6:**
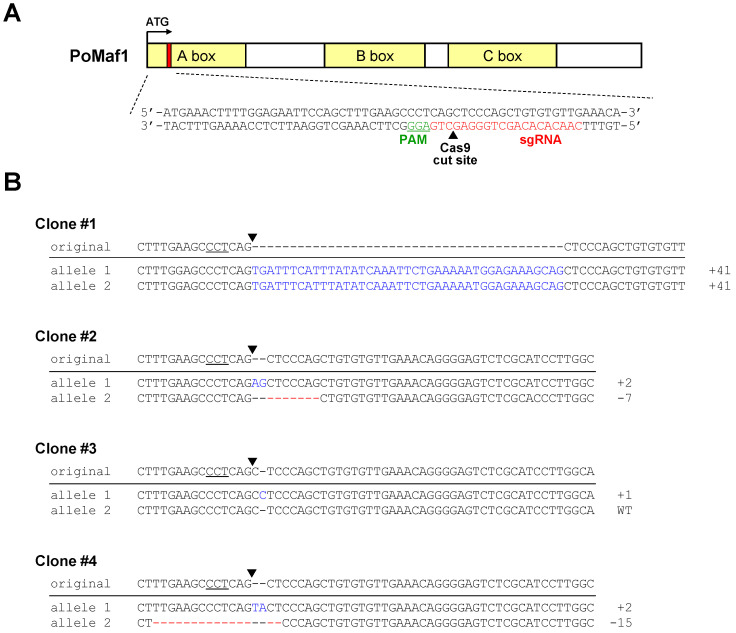
Construction of single cell-derived *PoMaf1* knockout (KO) clones using CRISPR/Cas9 system. (**A**) Simplified schematic of the Cas9/sgRNA-targeting site in the *PoMaf1* coding region. sgRNA and protospacer-adjacent motif (PAM) sequences are labeled in red and green, respectively. The Cas9 cutting site is indicated by a black arrow. (**B**) Indel sequences of mutant alleles of four single-cell-derived clones are shown compared to the original *PoMaf1* sequence. PAM sequences are underlined and the Cas9 cutting site is indicated by a black arrow. Insertions and deletions are indicated in blue and red, respectively. The size of each indel is indicated on the right. Genomic DNA of each clone was extracted and amplified by PCR. TA clones of the PCR products were subjected to Sanger sequencing.

To explore the function of *PoMaf1* in olive flounder HINAE cells, we knocked out *PoMaf1* using the CRISPR/Cas9 system to generate homogeneous mutant cell clones and overcome the low transfection efficiency of fish cell lines. Based on the *PoMaf1* genomic DNA sequence, the CRISPR target sequence was selected from several candidates to design a sgRNA (Figure 6A). The target sequence did not span an exon–exon junction and matched the *PoMaf1* genomic DNA sequence. A Cas9 plasmid (pX458) containing the U6 promoter-driven *PoMaf1* sgRNA expression cassette (pSpCas9(BB)-2A-GFP-PoMaf1 sgRNA) was transfected into HINAE cells and cells expressing GFP were sorted. The sequencing results of representative mutant clones are shown in Figure 6B. Clone #1, with a 41 bp insertion in both alleles, was a homozygous mutant clone. Clone #2, with a 2 bp insertion and 7 bp deletion in each allele, was a heterozygous biallelic mutant clone. Clone #3, with a 1 bp insertion in one allele, was a heterozygous monoallelic mutant clone. Clone #4, with a 2 bp insertion and 15 bp deletion in each allele, was a heterozygous biallelic mutant. Mutations of the 41 bp insertion, 2 bp insertion, 7 bp deletion, 1 bp insertion, and 2 bp insertion led to frameshift and nonsense mutations by out-of-frame indels, resulting in a shortened and possibly dysfunctional PoMAF1 protein. The 15 bp deletion mutation caused a non-frameshift mutation. Therefore, the CRISPR/Cas9 system enabled targeting and disruption of *PoMaf1* in HINAE cells.

### 2.6. PoMaf1 Disruption Enhances VHSV G mRNA Level in HINAE Cells

**Figure 7:**
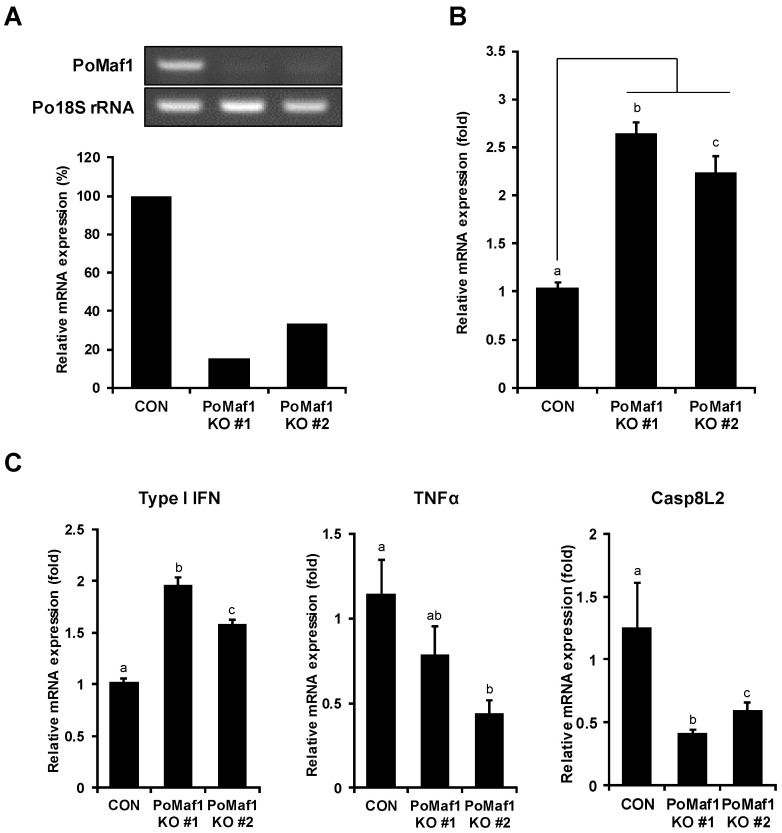
*PoMaf1* KO activates VHSV G expression in VHSV-infected cells. (**A**) Relative *PoMaf1* mRNA levels in control HINAE cells and *PoMaf1* KO cells by RT-PCR. RT-PCR band intensities were measured using ImageJ software (ver. 1.53 g; NIH, Bethesda, MD, USA). (**B**) Relative VHSV G mRNA levels in *PoMaf1* KO cells infected with VHSV. HINAE cells were infected with VHSV at a MOI of 0.5 and samples were collected at 48 h post infection. (**C**) Relative mRNA levels of type I IFN, TNFα and Caspase 8 like 2 (Casp8L2) in *PoMaf1* KO cells infected with VHSV. HINAE cells were infected with VHSV at a MOI of 0.5 and samples were collected at 48 h post infection. Target mRNA levels in clones #1 and #2 revealed by quantitative real-time PCR, as compared to the cells transfected with vector control (CON). Expression levels were normalized to that of 18S rRNA. Means and standard deviations of three independent replicates are shown. Means with the same superscript letter are not significantly different (*p* ≤ 0.05).

To determine the effect of *PoMaf1* KO on VHSV replication, *PoMaf1* KO cells were infected with VHSV, and viral gene expression was analyzed. Among the cell clones harboring indels in the *PoMaf1* locus, two biallelic mutant clones (clones #1 and #2) producing truncated PoMAF1 protein from both alleles were subjected to analysis of the *PoMaf1* mRNA level (Figure 7A). The *PoMaf1* RT-PCR primers were designed to span the indel junction of mutant alleles so that they bind only intact mRNA of *PoMaf1*. The *PoMaf1* transcript level was significantly reduced, by 85% and 67%, in *PoMaf1* KO clones #1 and #2, respectively, compared to control HINAE cells transfected with empty Cas9 vector (pX458). The higher *PoMaf1* mRNA level in *PoMaf1* KO clone #2 compared to clone #1 was a result of detection of the target PoMaf1 cDNA sequence with a 2 bp deletion in one allele. In *PoMaf1* KO clones #1 and #2, the VHSV G mRNA level increased 2.2 to 2.5-fold compared to control HINAE cells transfected with pX458, indicating that *PoMaf1* KO promoted VHSV infection of HINAE cells (Figure 7B). In addition, *PoMaf1* expression was inhibited by VHSV infection, and disruption of *PoMaf1* enhanced VHSV expression. Taken together, these results suggest that PoMAF1 inhibits VHSV, and that VHSV downregulates *PoMaf1* to promote its replication.

These observations raise the question as to how PoMAF1 regulates VHSV replication. Regarding the function of MAF1 as a repressor of RNA pol, several RNA viruses—including influenza virus and hepatitis delta virus—have been reported to require host RNA pol for their replication [4,39,40]. However, whether VHSV interacts with host RNA pol, and whether MAF1 suppresses viral RNA pol, is unclear. Production of DENV, another RNA virus, is regulated by Akt/FoxO1/Maf1 signaling [35]. In this study, a reduction in *PoMaf1* expression promoted VHSV expression; the underlying mechanism warrants further investigation.

RNA pol III activity is closely related to the immune response, suggesting that MAF1 may have a role in immunity [41,42]. MAF1 plays an important role in normal physiology and disease, and is involved in tumor immunity in colorectal cancer [5,43]. To investigate VHSV-induced immune response in *PoMaf1* KO cells, the transcription levels of innate immune response genes such as *type I IFN*, *TNFα* and *Caspase 8 like 2* (*Casp8L2*) were analyzed in *PoMaf1* KO cells (Figure 7C). The type I IFN mRNA level increased in both *PoMaf1* KO cells against VHSV infection, indicating that *PoMaf1* disruption led to increased IFN and VHSV G expression level. *IFN* is a major immune response gene induced in VHSV-infected *P. olivaceus* and HINAE cells, suggesting that *PoMaf1* is involved in immune response [44,45]. Focusing on the function of MAF1 as a repressor of RNA pol III, type I IFN is induced by RNA pol III through the RIG-I pathway [42], suggesting that *PoMaf1* disruption enhances the IFN expression level by activating RNA pol III. However, it is unclear how enhanced IFN levels matched increased VHSV G expression. Recently, it has been reported that a high-virulence VHSV strain induced higher levels of IFN 1 compared to a low-virulence VHSV strain, suggesting that the IFN response in VHSV infection correlates with virus titers but does not necessarily correlate with protection [45]. On the other hand, mRNA expression levels of pro-inflammatory cytokine TNFα and apoptosis initiator Casp8L2 decreased in *PoMaf1* KO cells during VHSV infection. In zebrafish, PoMAF1 overexpression upregulated the mRNA levels of key regulators of the innate immune response (unpublished data). Therefore, PoMAF1 is involved in the immune response to VHSV infection, although further studies are needed.

PoMAF1 is a potential therapeutic target VHSV. The CRISPR/Cas9 system enables genome editing and has revolutionized genetic improvement of fish, so we can apply it to upregulate *PoMaf1*. To enhance target gene expression, regulatory elements are inserted upstream of an endogenous gene by a CRISPR/Cas9-mediated knock-in strategy [46]. In contrast, *cis*-regulatory elements (CREs) capable of silencing a gene promoter region can be targeted by the CRISPR/Cas9 system without adding exogenous genes [47]. The CRISPR/Cas9 system in combination with multiple sgRNAs can cause large deletions in the target region [48]. CRISPR/Cas9-based genome editing has been accomplished in both *P. olivaceus*-derived cell lines and *P. olivaceus*, leading to investigation of gene function and improvement of the traits of *P. olivaceus* [22].

## 3. Materials and Methods

### 3.1. Fish and Sample Preparation

Olive flounder *Paralichthys olivaceus* were raised at the National Institute of Fisheries Sciences (NIFS; Busan, Korea) and maintained in 1-ton flow-through tanks at 19 ± 0.3 °C under a natural photoperiod using fluorescent lighting and an electronic timer (Yuwon Engineering Co., Hwaseong, Korea). Adults were fed to satiation with commercial extruded pellets (Daebong LS Co., Ltd., Jeju, Korea) twice daily to ensure a sufficient food supply. For RNA extraction, tissues were dissected from three healthy *P. olivaceus*, immediately frozen in liquid nitrogen, and stored at −80 °C before use. Animal experiments were conducted in accordance with the Animal Protection Act of the Republic of Korea, and approved by the Institutional Animal Care and Use Committee of the NIFS (2019-NIFS-IACUC-10, 1 February 2019).

### 3.2. Cloning and Sequencing of PoMaf1 from P. olivaceus

To amplify *PoMaf1*, the primers PoMaf1-ORF-F (5′-CAA TGA AAC TTT TGG AGA ATT CC-3′) and PoMaf1-ORF-R (5′-CAT CTC GAG TCA CAC GCA CAG CGC-3′) were designed based on *P. olivaceus* transcriptome analysis. The *P. olivaceus* cDNA library was used as the template for reverse transcription-polymerase chain reaction (PCR) to amplify the coding region of putative PoMaf1. The PCR fragments were purified using a gel extraction kit (Qiagen, Venlo, The Netherlands), and cloned into the pGEM^®^-T easy vector (Promega, Madison, WI, USA) according to the manufacturer’s instructions. Putative PoMaf1 was sequenced using the universal primer T7 promoter, SP6, on an ABI3730xl automatic sequencer (Applied Biosystems, Inc., Foster City, CA, USA). The *PoMaf1* sequence is deposited in GenBank with the accession number MW417124.

### 3.3. Amino Acid Sequence Analysis

The pI and MW of the deduced PoMAF1 protein were computed on the ExPASy (http://web.expasy.org/compute_pi/). The signal sequence was predicted using SignalP (http://www.cbs.dtu.dk/services/SignalP/). Disulfide bonds were predicted using DiANNA (http://clavius.bc.edu/~clotelab/DiANNA). Potential N-glycosylation sites were assessed using the NetNGlyc1.0 Server (http://www.cbs.dtu.dk/services/NetNGlyc/).

### 3.4. Multiple Alignment and Phylogenetic Analysis

Coding sequence homology was compared in BLASTX (http://www.ncbi.nlm.nih.gov/BLAST/). Multiple alignments were generated with CLUSTALW (http://www.genome.jp/tools-bin/clustalw) and used for assessing similarities among the aligned sequences with MEGA software (ver. 5; Arizona State University, AZ, USA; [49]). An unrooted phylogenetic tree based on the deduced amino acid sequences was constructed using the neighbor-joining method. The bootstrap resampling was repeated 1000 times to determine the reliability of the phylogenetic tree.

### 3.5. RNA Isolation, Reverse Transcriptase PCR and Quantitative Real-Time PCR

Total RNA was isolated using TRIzol™ reagent (Invitrogen, Carlsbad, CA, USA) according to the manufacturer’s instructions, and treated with DNase (New England BioLabs, Beverly, MA, USA). RNA samples (500 ng) were used to synthesize first-strand cDNA with the Transcriptor First Strand cDNA Synthesis Kit (Roche, Basel, Switzerland). RT-PCR was performed using Ex Taq™ polymerase (TaKaRa Bio Inc., Shiga, Japan) and gene-specific primers: PoMaf1-F (5′-AGC CCT CAG CTC CCA GCT-3′), PoMaf1-R (5′-GTC CTG GAG GCT CTT TCT CC-3′), Po18S-RT-F (5′-ATG GCC GTT CTT AGT TGG TG-3′) and Po18S-RT-R (5′-CAC ACG CTG ATC CAG TCA GT-3′). The PCR reaction conditions were denaturation for 5 min at 95 °C; followed by 25 or 35 cycles of 95 °C for 30 s, 60 °C for 30 s, and 95 °C for 30 s; and a final extension at 72 °C for 10 min. Quantitative real-time PCR was carried out using Fast SYBR Green Master Mix (Applied Biosystems, Inc.) and the following primers for *PoMaf1*, VHSV *G*, *type I IFN*, *TNFα* and *Casp8L2*: PoMaf1-RT-F (5′-GGG AAA ACC CTC TGA GTG ACA A-3′), PoMaf1-RT-R (5′-CGC CGC ACT GAA GTC ATA GTC-3′), VHSV-G-RT-F (5′-AGA TGA GGG GAG CCA CAG AC-3′), VHSV-G-RT-R (5′-GGG ATG ATC AAT TTG TCC CC-3′), PoIFN1-RT-F (5′-GGC CAC ATT CAC GCA ATC AC-3′), PoIFN2-RT-R (5′-TGC AGG TGT CTA TGT GGC TA-3′), PoTNFα-RT-F (5′-CCG ACT GGA TGT GTA AGG TG-3′), PoTNFα-RT-R (5′-GTT GTG GGG TTC TGT TTT CTC-3′), PoCasp8L2-RT-F (5′-ACC ACG TCT TCC ATG AGA CC-3′), PoCasp8L3-RT-R (5′-GCC CAG CCA CTT AAA CAC AT-3′). The primers for *P. olivaceus* 18S rRNA were the same as those for RT-PCR. Amplification and detection were performed using the ABI 7500 Real-Time PCR System (Applied Biosystems, Inc.) with the following steps: 50 °C for 2 min, 95 °C for 10 min, and 40 cycles of 95 °C for 15 s and 60 °C for 30 s. Quantitative real-time PCR data were analyzed using the comparative threshold cycle (Ct) method (2^−ΔΔCT^ method) for relative quantification.

### 3.6. sgRNA Design and Vector Construction for the CRISPR/Cas9 System

Using the CRISPR RGEN Tools (www.rgenome.net/cas-designer), a single guide RNA (sgRNA) targeting *PoMaf1* was designed (5′-CAA CAC ACA GCT GGG AGC TGA GG-3′; PAM, underlined). A pair of oligonucleotides corresponding to the target sgRNA sequence (oligo-1, 5′-CAC CGC AAC ACA CAG CTG GGA GCT G-3′; oligo-2, 5′-AAA CCA GCT CCC AGC TGT GTG TTG-3′) was inserted into the sgRNA/Cas9 dual expression vector pSpCas9(BB)-2A-GFP (also known as PX458; Addgene, Watertown, MA, USA) according to the protocol of Zhang [50]. Briefly, the pX458 vector was digested with BbsI and gel-purified. The dsDNA with 4 bp overhangs on both ends was generated by annealing following phosphorylation, and ligated into the linearized pX458 vector.

### 3.7. Cell Culture and Transfection

HINAE olive flounder embryonic cells, a gift from Takashi Aoki, were maintained in Leibovitz’s L-15 medium (L-15; Gibco, Invitrogen, Carlsbad, CA, USA) with 10% heat-inactivated fetal bovine serum (FBS; Gibco) and 1% (*v*/*v*) antibiotic-antimycotic (AA; Gibco) at 20 °C. Medium was changed every third day. HINAE cells were transfected with ViaFect™ transfection reagent (Promega) according to the manufacturer’s instructions.

### 3.8. Production of Single-Cell Clones of KO Cells

To obtain single *PoMaf1* KO cells, HINAE cells were transfected with Cas9/*PoMaf1* sgRNA and EGFP co-expression vector (pSpCas9(BB)-2A-GFP-PoMaf1 sgRNA). At 48 h post transfection, cells were trypsinized for 3 min at room temperature and collected by centrifugation. Cells were resuspended in L-15 culture medium containing 15% heat-inactivated FBS and 1.5% (*v*/*v*) AA before being passed through a 35 μm cell strainer into tubes (BD Falcon, Franklin Lakes, NJ, USA). Cells positive for GFP (and therefore Cas9) expression were sorted and transferred to 96-well plates using a cell sorter (BD FACS Aria III; BD Biosciences, San Jose, CA, USA) following the manufacturer’s instructions. Control cells were transfected with the empty pX458 construct. After culture for one month, genomic DNA was extracted from the clones using PrimePrep Direct PCR Reagent (GeNetBio, Daejeon, Korea) following the manufacturer’s instructions, and the fragments containing the CRISPR target site were amplified by PCR using specific primers (PoMaf1-seq-F, 5′-TGT TTT GCA AGG TGA CTG TAC GT-3′; PoMaf1-seq-R, 5′-GGC GTG ACT TTT GTT GAG TAT TAA CT-3′). The PCR amplicons were purified and cloned into the pGEM^®^-T easy vector (Promega), and at least eight colonies per clone were sequenced to detect indels using an ABI3730xl Automatic Sequencer (Applied Biosystems, Inc.).

### 3.9. VHSV Preparation

VHSV isolated from a diseased *P. olivaceus* was propagated in a monolayer of HINAE cells at 20 °C [51]. When cytopathic effects became evident, the supernatants of VHSV-infected cell cultures were clarified by centrifugation and stored as aliquots at −80 °C. The tissue culture infective dose 50 (TCID50) was determined using the end-point dilution method in 96-well plates.

### 3.10. Statistical Analysis

All experiments were conducted using three biological replicates and results were represented as means ± standard deviation (SD) (*n* = 3). In Figure 4, Figure 5 and Figure 7, for a comparison of more than two groups, data were analyzed using one-way analysis of variance (ANOVA), followed by Duncan’s multiple range test. Statistical significance was considered at *p* ≤ 0.05. Statistical analyses were conducted using PASW Statistics (ver. 18.0; SPSS Inc., Chicago, IL, USA).

## 4. Conclusions

We characterized the full-length ORF of *Maf1* in *P. olivaceus* (*PoMaf1*). Deduced amino acids of PoMAF1 had putative A, B, C, box and MAF1 signature sequences comparable with MAF1 orthologues from other vertebrate species. The *PoMaf1* mRNA level was highest in eye tissue in *P. olivaceus* and markedly increased during early development. The *PoMaf1* mRNA level in HINAE cells decreased during VHSV infection. To investigate the role of PoMAF1 in VHSV infection, single-cell-derived *PoMaf1* KO cells were generated using the CRISPR/Cas9 system. In the absence of PoMAF1, the VHSV G mRNA level increased during VHSV infection, suggesting PoMAF1 inhibits VHSV replication. In addition, the VHSV-induced immune response in *PoMaf1* KO cells was investigated. This study provides fundamental data on fish *Maf1* and will facilitate the development of novel therapeutic approaches to VHSV infection. However, the mechanism underlying the inhibitory effect of PoMAF1 on VHSV infection warrants further investigation.

## Figures and Tables

**Table 1:** GenBank accession numbers used for phylogenetic tree construction.

Species	Accession No.	Species	Accession No.
*Seriola dumerili*	XP_022622762.1	*Oryza sativa*	XP_015636424.1
*Scophthalmus maximus*	XP_035474856.1	*Drosophila melanogaster*	NP_001015167.2
*Homo sapiens*	AAH14082.1	*Aspergillus niger*	PYH58464.1
*Danio rerio*	NP_001025410.1	*Citrus sinensis*	NP_001275774.1
*Mus musculus*	AAH16260.1	*Upupa epops*	NWU98758.1
*Mesembrinibis cayennensis*	NXL10327.1	*Glaucidium brasilianum*	NXL38974.1
*Platysternon megacephalum*	TFJ99060.1	*Microcaecilia unicolor*	XP_030076765.1
*Salmo salar*	ACI33843.1	*Xenopus laevis*	NP_001080708.1
*Saccharomyces cerevisiae*	QHB07477.1	*Gopherus evgoodei*	XP_030409929.1
*Trypanosoma brucei*	RHW73349.1	*Chelonia mydas*	XP_027690307.1
*Colletotrichum orbiculare*	TDZ26909.1	*Monomorium pharaonis*	XP_012522189.1
*Arabidopsis lyrata* (MAF1 isoform X1)	XP_020878685.1	*Callosobruchus maculatus*	VEN63387.1
*Arabidopsis lyrata* (MAF1 isoform X2)	XP_020878686.1	*Daphnia magna*	XP_032783504.1
*Sus scrofa*	XP_003125492.2	*Crassostrea gigas*	XP_011417596.2
*Pipistrellus kuhlii*	KAF6276919.1	*Mytilus coruscus*	CAC5403110.1

## Data Availability

The data presented in this study are available in this article.
